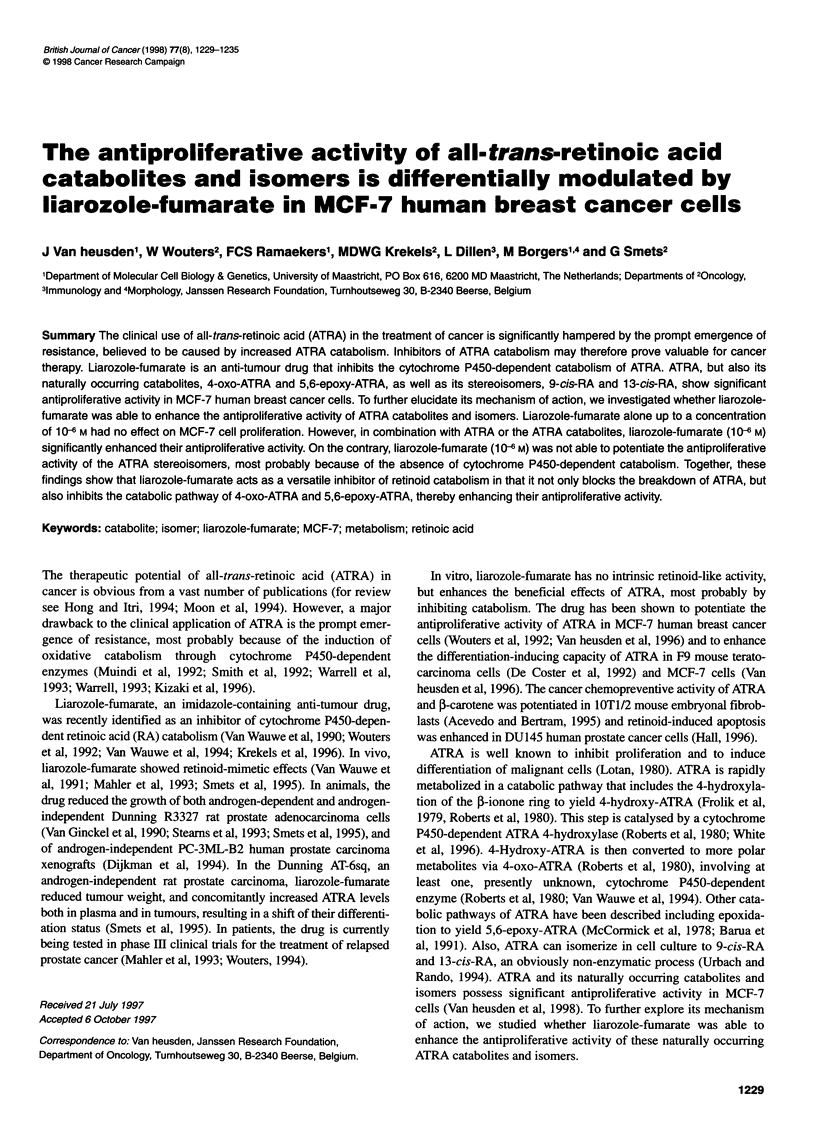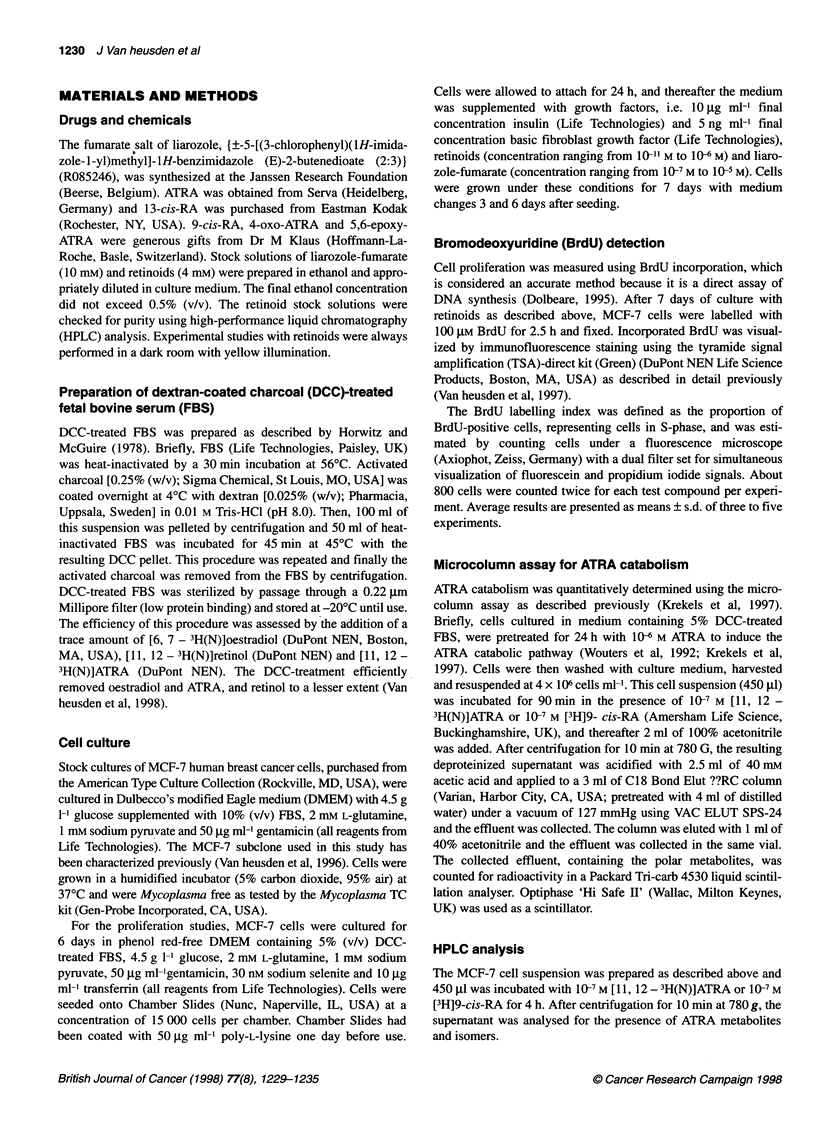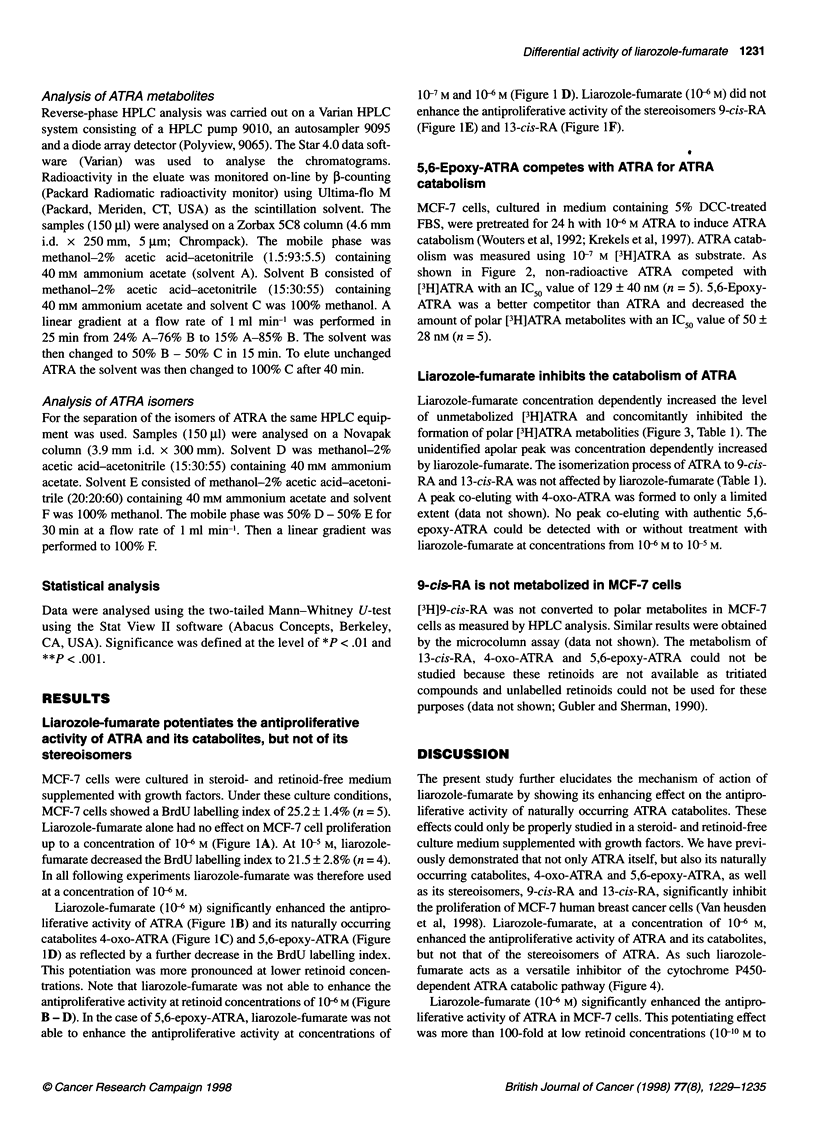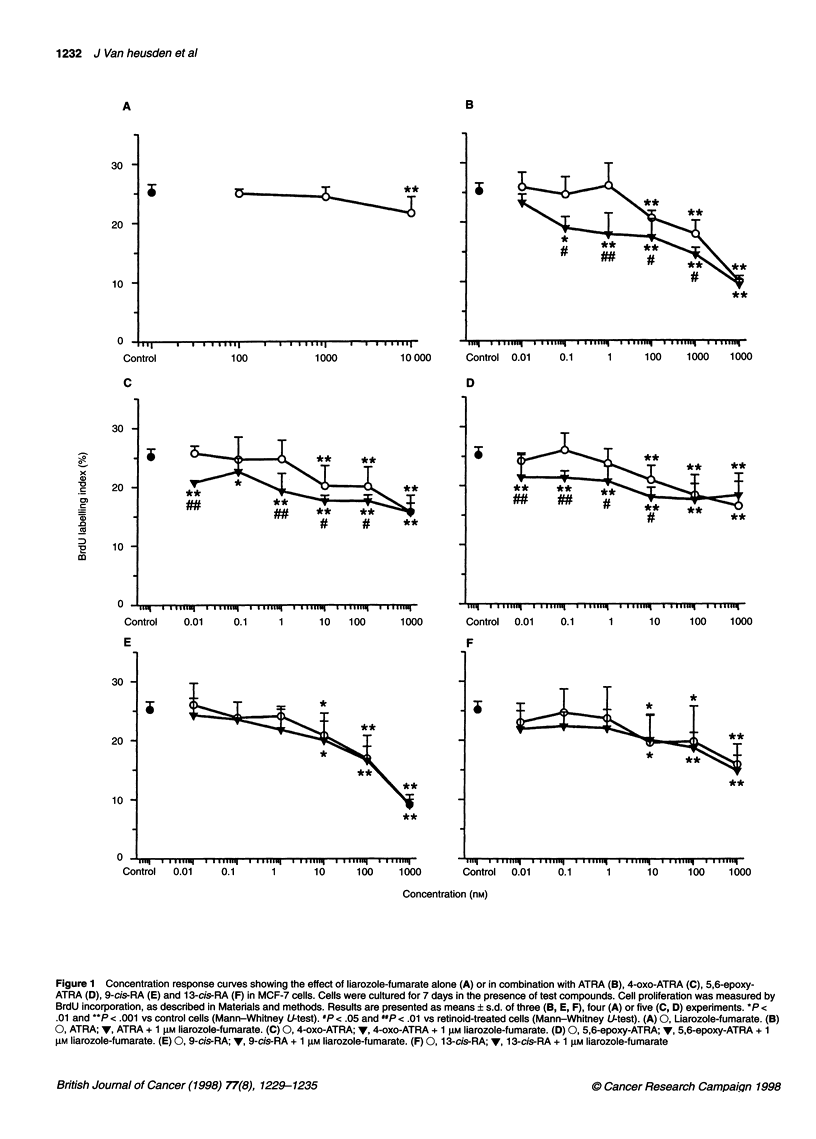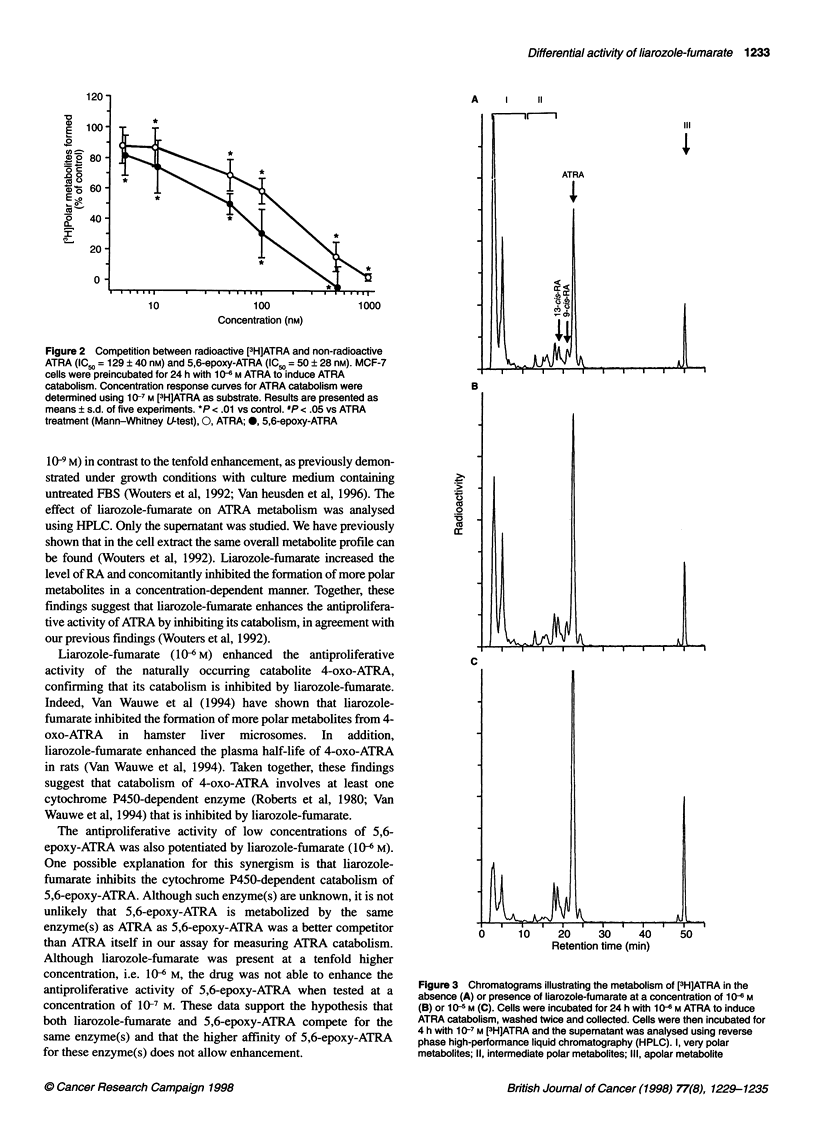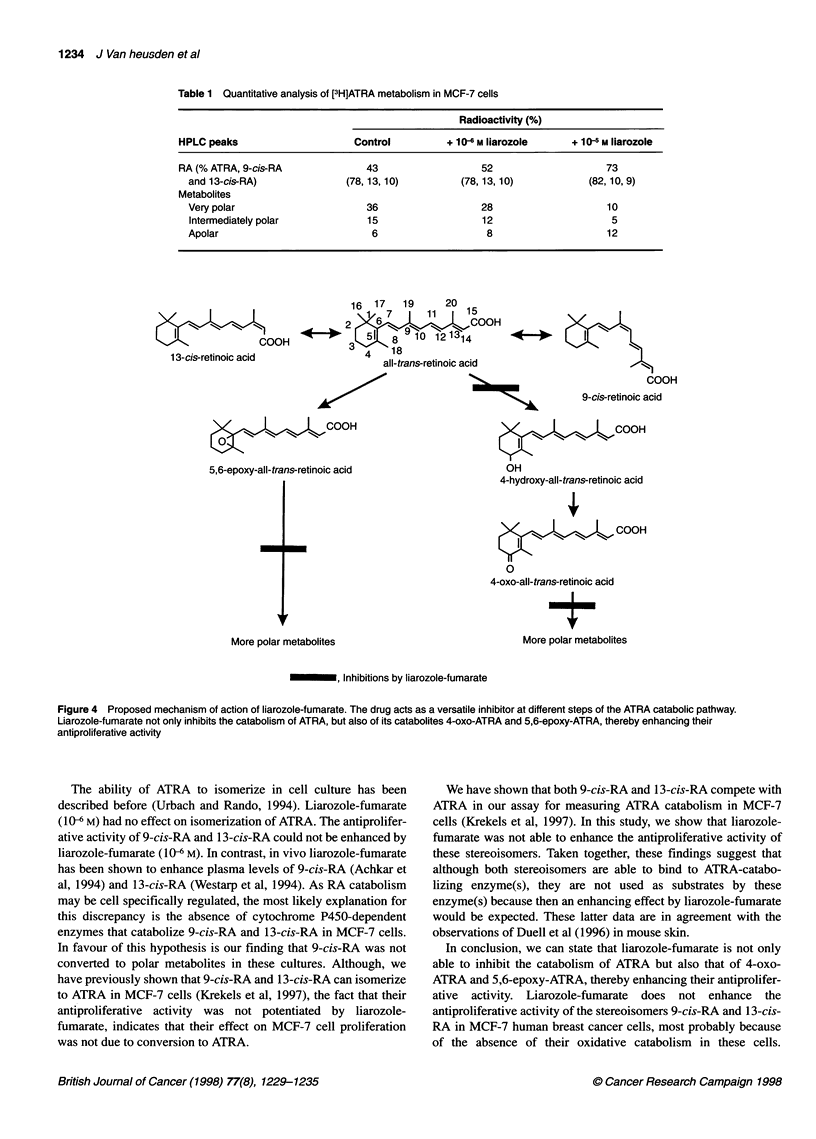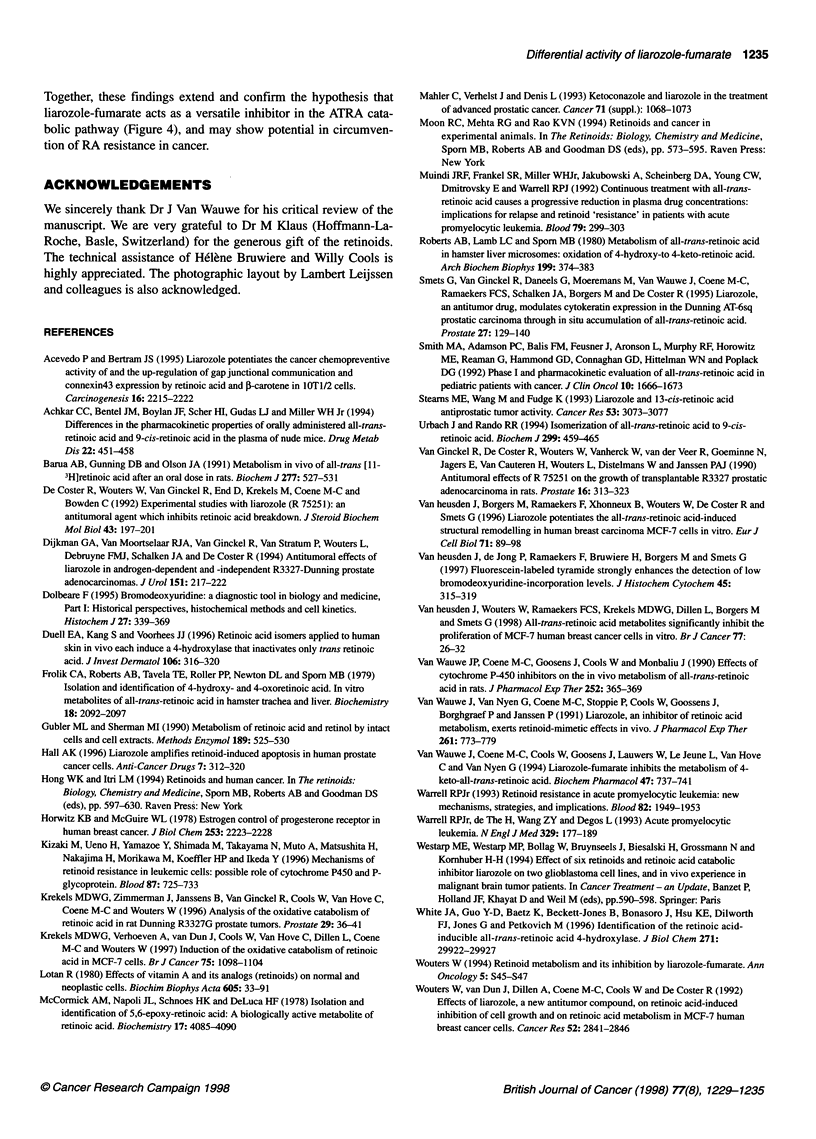# The antiproliferative activity of all-trans-retinoic acid catabolites and isomers is differentially modulated by liarozole-fumarate in MCF-7 human breast cancer cells.

**DOI:** 10.1038/bjc.1998.207

**Published:** 1998-04

**Authors:** J. Van heusden, W. Wouters, F. C. Ramaekers, M. D. Krekels, L. Dillen, M. Borgers, G. Smets

**Affiliations:** Department of Molecular Cell Biology & Genetics, University of Maastricht, The Netherlands.

## Abstract

The clinical use of all-trans-retinoic acid (ATRA) in the treatment of cancer is significantly hampered by the prompt emergence of resistance, believed to be caused by increased ATRA catabolism. Inhibitors of ATRA catabolism may therefore prove valuable for cancer therapy. Liarozole-fumarate is an anti-tumour drug that inhibits the cytochrome P450-dependent catabolism of ATRA. ATRA, but also its naturally occurring catabolites, 4-oxo-ATRA and 5,6-epoxy-ATRA, as well as its stereoisomers, 9-cis-RA and 13-cis-RA, show significant antiproliferative activity in MCF-7 human breast cancer cells. To further elucidate its mechanism of action, we investigated whether liarozole-fumarate was able to enhance the antiproliferative activity of ATRA catabolites and isomers. Liarozole-fumarate alone up to a concentration of 10(-6) M had no effect on MCF-7 cell proliferation. However, in combination with ATRA or the ATRA catabolites, liarozole-fumarate (10(-6) M) significantly enhanced their antiproliferative activity. On the contrary, liarozole-fumarate (10(-6) M) was not able to potentiate the antiproliferative activity of the ATRA stereoisomers, most probably because of the absence of cytochrome P450-dependent catabolism. Together, these findings show that liarozole-fumarate acts as a versatile inhibitor of retinoid catabolism in that it not only blocks the breakdown of ATRA, but also inhibits the catabolic pathway of 4-oxo-ATRA and 5,6-epoxy-ATRA, thereby enhancing their antiproliferative activity.


British Journal of Cancer (1998) 77(8), 1229–1235
© 1998 Cancer Research Campaign

### The antiproliferative activity of all-*trans*-retinoic acid catabolites and isomers is differentially modulated by liarozole-fumarate in MCF-7 human breast cancer cells

**J Van heusden[1], W Wouters[2], FCS Ramaekers[1], MDWG Krekels[2], L Dillen[3], M Borgers[1,4] and G Smets[2]**

[1]Department of Molecular Cell Biology & Genetics, University of Maastricht, PO Box 616, 6200 MD Maastricht, The Netherlands; Departments of [2]Oncology, [3]Immunology and [4]Morphology, Janssen Research Foundation, Turnhoutseweg 30, B-2340 Beerse, Belgium

**Summary** The clinical use of all-*trans*-retinoic acid (ATRA) in the treatment of cancer is significantly hampered by the prompt emergence of resistance, believed to be caused by increased ATRA catabolism. Inhibitors of ATRA catabolism may therefore prove valuable for cancer therapy. Liarozole-fumarate is an anti-tumour drug that inhibits the cytochrome P450-dependent catabolism of ATRA. ATRA, but also its naturally occurring catabolites, 4-oxo-ATRA and 5,6-epoxy-ATRA, as well as its stereoisomers, 9-*cis*-RA and 13-*cis*-RA, show significant antiproliferative activity in MCF-7 human breast cancer cells. To further elucidate its mechanism of action, we investigated whether liarozole-fumarate was able to enhance the antiproliferative activity of ATRA catabolites and isomers. Liarozole-fumarate alone up to a concentration of $10^{-6}$ M had no effect on MCF-7 cell proliferation. However, in combination with ATRA or the ATRA catabolites, liarozole-fumarate ($10^{-6}$ M) significantly enhanced their antiproliferative activity. On the contrary, liarozole-fumarate ($10^{-6}$ M) was not able to potentiate the antiproliferative activity of the ATRA stereoisomers, most probably because of the absence of cytochrome P450-dependent catabolism. Together, these findings show that liarozole-fumarate acts as a versatile inhibitor of retinoid catabolism in that it not only blocks the breakdown of ATRA, but also inhibits the catabolic pathway of 4-oxo-ATRA and 5,6-epoxy-ATRA, thereby enhancing their antiproliferative activity.

**Keywords:** catabolite; isomer; liarozole-fumarate; MCF-7; metabolism; retinoic acid

The therapeutic potential of all-*trans*-retinoic acid (ATRA) in cancer is obvious from a vast number of publications (for review see Hong and Itri, 1994; Moon et al, 1994). However, a major drawback to the clinical application of ATRA is the prompt emergence of resistance, most probably because of the induction of oxidative catabolism through cytochrome P450-dependent enzymes (Muindi et al, 1992; Smith et al, 1992; Warrell et al, 1993; Warrell, 1993; Kizaki et al, 1996).

Liarozole-fumarate, an imidazole-containing anti-tumour drug, was recently identified as an inhibitor of cytochrome P450-dependent retinoic acid (RA) catabolism (Van Wauwe et al, 1990; Wouters et al, 1992; Van Wauwe et al, 1994; Krekels et al, 1996). In vivo, liarozole-fumarate showed retinoid-mimetic effects (Van Wauwe et al, 1991; Mahler et al, 1993; Smets et al, 1995). In animals, the drug reduced the growth of both androgen-dependent and androgen-independent Dunning R3327 rat prostate adenocarcinoma cells (Van Ginckel et al, 1990; Stearns et al, 1993; Smets et al, 1995), and of androgen-independent PC-3ML-B2 human prostate carcinoma xenografts (Dijkman et al, 1994). In the Dunning AT-6sq, an androgen-independent rat prostate carcinoma, liarozole-fumarate reduced tumour weight, and concomitantly increased ATRA levels both in plasma and in tumours, resulting in a shift of their differentiation status (Smets et al, 1995). In patients, the drug is currently being tested in phase III clinical trials for the treatment of relapsed prostate cancer (Mahler et al, 1993; Wouters, 1994).

*Received 21 July 1997*
*Accepted 6 October 1997*

*Correspondence to:* Van heusden, Janssen Research Foundation, Department of Oncology, Turnhoutseweg 30, B-2340 Beerse, Belgium.

In vitro, liarozole-fumarate has no intrinsic retinoid-like activity, but enhances the beneficial effects of ATRA, most probably by inhibiting catabolism. The drug has been shown to potentiate the antiproliferative activity of ATRA in MCF-7 human breast cancer cells (Wouters et al, 1992; Van heusden et al, 1996) and to enhance the differentiation-inducing capacity of ATRA in F9 mouse teratocarcinoma cells (De Coster et al, 1992) and MCF-7 cells (Van heusden et al, 1996). The cancer chemopreventive activity of ATRA and β-carotene was potentiated in 10T1/2 mouse embryonal fibroblasts (Acevedo and Bertram, 1995) and retinoid-induced apoptosis was enhanced in DU145 human prostate cancer cells (Hall, 1996).

ATRA is well known to inhibit proliferation and to induce differentiation of malignant cells (Lotan, 1980). ATRA is rapidly metabolized in a catabolic pathway that includes the 4-hydroxylation of the β-ionone ring to yield 4-hydroxy-ATRA (Frolik et al, 1979, Roberts et al, 1980). This step is catalysed by a cytochrome P450-dependent ATRA 4-hydroxylase (Roberts et al, 1980; White et al, 1996). 4-Hydroxy-ATRA is then converted to more polar metabolites via 4-oxo-ATRA (Roberts et al, 1980), involving at least one, presently unknown, cytochrome P450-dependent enzyme (Roberts et al, 1980; Van Wauwe et al, 1994). Other catabolic pathways of ATRA have been described including epoxidation to yield 5,6-epoxy-ATRA (McCormick et al, 1978; Barua et al, 1991). Also, ATRA can isomerize in cell culture to 9-*cis*-RA and 13-*cis*-RA, an obviously non-enzymatic process (Urbach and Rando, 1994). ATRA and its naturally occurring catabolites and isomers possess significant antiproliferative activity in MCF-7 cells (Van heusden et al, 1998). To further explore its mechanism of action, we studied whether liarozole-fumarate was able to enhance the antiproliferative activity of these naturally occurring ATRA catabolites and isomers.

### MATERIALS AND METHODS

### Drugs and chemicals

The fumarate salt of liarozole, {±-5-[(3-chlorophenyl)(1*H*-imidazole-1-yl)methyl]-1*H*-benzimidazole (E)-2-butenedioate (2:3)} (R085246), was synthesized at the Janssen Research Foundation (Beerse, Belgium). ATRA was obtained from Serva (Heidelberg, Germany) and 13-*cis*-RA was purchased from Eastman Kodak (Rochester, NY, USA). 9-*cis*-RA, 4-oxo-ATRA and 5,6-epoxy-ATRA were generous gifts from Dr M Klaus (Hoffmann-La-Roche, Basle, Switzerland). Stock solutions of liarozole-fumarate (10 mM) and retinoids (4 mM) were prepared in ethanol and appropriately diluted in culture medium. The final ethanol concentration did not exceed 0.5% (v/v). The retinoid stock solutions were checked for purity using high-performance liquid chromatography (HPLC) analysis. Experimental studies with retinoids were always performed in a dark room with yellow illumination.

### Preparation of dextran-coated charcoal (DCC)-treated fetal bovine serum (FBS)

DCC-treated FBS was prepared as described by Horwitz and McGuire (1978). Briefly, FBS (Life Technologies, Paisley, UK) was heat-inactivated by a 30 min incubation at 56°C. Activated charcoal [0.25% (w/v); Sigma Chemical, St Louis, MO, USA] was coated overnight at 4°C with dextran [0.025% (w/v); Pharmacia, Uppsala, Sweden] in 0.01 M Tris-HCl (pH 8.0). Then, 100 ml of this suspension was pelleted by centrifugation and 50 ml of heat-inactivated FBS was incubated for 45 min at 45°C with the resulting DCC pellet. This procedure was repeated and finally the activated charcoal was removed from the FBS by centrifugation. DCC-treated FBS was sterilized by passage through a 0.22 μm Millipore filter (low protein binding) and stored at –20°C until use. The efficiency of this procedure was assessed by the addition of a trace amount of [6, 7 – $^{3}$H(N)]oestradiol (DuPont NEN, Boston, MA, USA), [11, 12 – $^{3}$H(N)]retinol (DuPont NEN) and [11, 12 – $^{3}$H(N)]ATRA (DuPont NEN). The DCC-treatment efficiently removed oestradiol and ATRA, and retinol to a lesser extent (Van heusden et al, 1998).

### Cell culture

Stock cultures of MCF-7 human breast cancer cells, purchased from the American Type Culture Collection (Rockville, MD, USA), were cultured in Dulbecco's modified Eagle medium (DMEM) with 4.5 g $l^{-1}$ glucose supplemented with 10% (v/v) FBS, 2 mM L-glutamine, 1 mM sodium pyruvate and 50 μg $ml^{-1}$ gentamicin (all reagents from Life Technologies). The MCF-7 subclone used in this study has been characterized previously (Van heusden et al, 1996). Cells were grown in a humidified incubator (5% carbon dioxide, 95% air) at 37°C and were *Mycoplasma* free as tested by the *Mycoplasma* TC kit (Gen-Probe Incorporated, CA, USA).

For the proliferation studies, MCF-7 cells were cultured for 6 days in phenol red-free DMEM containing 5% (v/v) DCC-treated FBS, 4.5 g $l^{-1}$ glucose, 2 mM L-glutamine, 1 mM sodium pyruvate, 50 μg $ml^{-1}$gentamicin, 30 nM sodium selenite and 10 μg $ml^{-1}$ transferrin (all reagents from Life Technologies). Cells were seeded onto Chamber Slides (Nunc, Naperville, IL, USA) at a concentration of 15 000 cells per chamber. Chamber Slides had been coated with 50 μg $ml^{-1}$ poly-L-lysine one day before use. Cells were allowed to attach for 24 h, and thereafter the medium was supplemented with growth factors, i.e. 10 μg $ml^{-1}$ final concentration insulin (Life Technologies) and 5 ng $ml^{-1}$ final concentration basic fibroblast growth factor (Life Technologies), retinoids (concentration ranging from $10^{-11}$ M to $10^{-6}$ M) and liarozole-fumarate (concentration ranging from $10^{-7}$ M to $10^{-5}$ M). Cells were grown under these conditions for 7 days with medium changes 3 and 6 days after seeding.

### Bromodeoxyuridine (BrdU) detection

Cell proliferation was measured using BrdU incorporation, which is considered an accurate method because it is a direct assay of DNA synthesis (Dolbeare, 1995). After 7 days of culture with retinoids as described above, MCF-7 cells were labelled with 100 μM BrdU for 2.5 h and fixed. Incorporated BrdU was visualized by immunofluorescence staining using the tyramide signal amplification (TSA)-direct kit (Green) (DuPont NEN Life Science Products, Boston, MA, USA) as described in detail previously (Van heusden et al, 1997).

The BrdU labelling index was defined as the proportion of BrdU-positive cells, representing cells in S-phase, and was estimated by counting cells under a fluorescence microscope (Axiophot, Zeiss, Germany) with a dual filter set for simultaneous visualization of fluorescein and propidium iodide signals. About 800 cells were counted twice for each test compound per experiment. Average results are presented as means ± s.d. of three to five experiments.

### Microcolumn assay for ATRA catabolism

ATRA catabolism was quantitatively determined using the microcolumn assay as described previously (Krekels et al, 1997). Briefly, cells cultured in medium containing 5% DCC-treated FBS, were pretreated for 24 h with $10^{-6}$ M ATRA to induce the ATRA catabolic pathway (Wouters et al, 1992; Krekels et al, 1997). Cells were then washed with culture medium, harvested and resuspended at 4 × $10^{6}$ cells $ml^{-1}$. This cell suspension (450 μl) was incubated for 90 min in the presence of $10^{-7}$ M [11, 12 – $^{3}$H(N)]ATRA or $10^{-7}$ M [$^{3}$H]9- *cis*-RA (Amersham Life Science, Buckinghamshire, UK), and thereafter 2 ml of 100% acetonitrile was added. After centrifugation for 10 min at 780 G, the resulting deproteinized supernatant was acidified with 2.5 ml of 40 mM acetic acid and applied to a 3 ml of C18 Bond Elut ??RC column (Varian, Harbor City, CA, USA; pretreated with 4 ml of distilled water) under a vacuum of 127 mmHg using VAC ELUT SPS-24 and the effluent was collected. The column was eluted with 1 ml of 40% acetonitrile and the effluent was collected in the same vial. The collected effluent, containing the polar metabolites, was counted for radioactivity in a Packard Tri-carb 4530 liquid scintillation analyser. Optiphase 'Hi Safe II' (Wallac, Milton Keynes, UK) was used as a scintillator.

### HPLC analysis

The MCF-7 cell suspension was prepared as described above and 450 μl was incubated with $10^{-7}$ M [11, 12 – $^{3}$H(N)]ATRA or $10^{-7}$ M [$^{3}$H]9-*cis*-RA for 4 h. After centrifugation for 10 min at 780 ***g***, the supernatant was analysed for the presence of ATRA metabolites and isomers.

*© Cancer Research Campaign 1998*

### *Analysis of ATRA metabolites*

Reverse-phase HPLC analysis was carried out on a Varian HPLC system consisting of a HPLC pump 9010, an autosampler 9095 and a diode array detector (Polyview, 9065). The Star 4.0 data software (Varian) was used to analyse the chromatograms. Radioactivity in the eluate was monitored on-line by β-counting (Packard Radiomatic radioactivity monitor) using Ultima-flo M (Packard, Meriden, CT, USA) as the scintillation solvent. The samples (150 μl) were analysed on a Zorbax 5C8 column (4.6 mm i.d. × 250 mm, 5 μm; Chrompack). The mobile phase was methanol–2% acetic acid–acetonitrile (1.5:93:5.5) containing 40 mM ammonium acetate (solvent A). Solvent B consisted of methanol–2% acetic acid–acetonitrile (15:30:55) containing 40 mM ammonium acetate and solvent C was 100% methanol. A linear gradient at a flow rate of 1 ml $min^{-1}$ was performed in 25 min from 24% A–76% B to 15% A–85% B. The solvent was then changed to 50% B – 50% C in 15 min. To elute unchanged ATRA the solvent was then changed to 100% C after 40 min.

### *Analysis of ATRA isomers*

For the separation of the isomers of ATRA the same HPLC equipment was used. Samples (150 μl) were analysed on a Novapak column (3.9 mm i.d. × 300 mm). Solvent D was methanol–2% acetic acid–acetonitrile (15:30:55) containing 40 mM ammonium acetate. Solvent E consisted of methanol–2% acetic acid–acetonitrile (20:20:60) containing 40 mM ammonium acetate and solvent F was 100% methanol. The mobile phase was 50% D – 50% E for 30 min at a flow rate of 1 ml $min^{-1}$. Then a linear gradient was performed to 100% F.

### Statistical analysis

Data were analysed using the two-tailed Mann–Whitney *U*-test using the Stat View II software (Abacus Concepts, Berkeley, CA, USA). Significance was defined at the level of $*P < .01$ and $**P < .001$.

### RESULTS

### Liarozole-fumarate potentiates the antiproliferative activity of ATRA and its catabolites, but not of its stereoisomers

MCF-7 cells were cultured in steroid- and retinoid-free medium supplemented with growth factors. Under these culture conditions, MCF-7 cells showed a BrdU labelling index of 25.2 ± 1.4% ($n = 5$). Liarozole-fumarate alone had no effect on MCF-7 cell proliferation up to a concentration of $10^{-6}$ M (Figure 1**A**). At $10^{-5}$ M, liarozole-fumarate decreased the BrdU labelling index to 21.5 ± 2.8% ($n = 4$). In all following experiments liarozole-fumarate was therefore used at a concentration of $10^{-6}$ M.

Liarozole-fumarate ($10^{-6}$ M) significantly enhanced the antiproliferative activity of ATRA (Figure 1**B**) and its naturally occurring catabolites 4-oxo-ATRA (Figure 1**C**) and 5,6-epoxy-ATRA (Figure 1**D**) as reflected by a further decrease in the BrdU labelling index. This potentiation was more pronounced at lower retinoid concentrations. Note that liarozole-fumarate was not able to enhance the antiproliferative activity at retinoid concentrations of $10^{-6}$ M (Figure **B** – **D**). In the case of 5,6-epoxy-ATRA, liarozole-fumarate was not able to enhance the antiproliferative activity at concentrations of $10^{-7}$ M and $10^{-6}$ M (Figure 1 **D**). Liarozole-fumarate ($10^{-6}$ M) did not enhance the antiproliferative activity of the stereoisomers 9-*cis*-RA (Figure 1**E**) and 13-*cis*-RA (Figure 1**F**).

### 5,6-Epoxy-ATRA competes with ATRA for ATRA catabolism

MCF-7 cells, cultured in medium containing 5% DCC-treated FBS, were pretreated for 24 h with $10^{-6}$ M ATRA to induce ATRA catabolism (Wouters et al, 1992; Krekels et al, 1997). ATRA catabolism was measured using $10^{-7}$ M [$^3$H]ATRA as substrate. As shown in Figure 2, non-radioactive ATRA competed with [$^3$H]ATRA with an $IC_{50}$ value of 129 ± 40 nM ($n = 5$). 5,6-Epoxy-ATRA was a better competitor than ATRA and decreased the amount of polar [$^3$H]ATRA metabolites with an $IC_{50}$ value of 50 ± 28 nM ($n = 5$).

### Liarozole-fumarate inhibits the catabolism of ATRA

Liarozole-fumarate concentration dependently increased the level of unmetabolized [$^3$H]ATRA and concomitantly inhibited the formation of polar [$^3$H]ATRA metabolities (Figure 3, Table 1). The unidentified apolar peak was concentration dependently increased by liarozole-fumarate. The isomerization process of ATRA to 9-*cis*-RA and 13-*cis*-RA was not affected by liarozole-fumarate (Table 1). A peak co-eluting with 4-oxo-ATRA was formed to only a limited extent (data not shown). No peak co-eluting with authentic 5,6-epoxy-ATRA could be detected with or without treatment with liarozole-fumarate at concentrations from $10^{-6}$ M to $10^{-5}$ M.

### 9-*cis*-RA is not metabolized in MCF-7 cells

[$^3$H]9-*cis*-RA was not converted to polar metabolites in MCF-7 cells as measured by HPLC analysis. Similar results were obtained by the microcolumn assay (data not shown). The metabolism of 13-*cis*-RA, 4-oxo-ATRA and 5,6-epoxy-ATRA could not be studied because these retinoids are not available as tritiated compounds and unlabelled retinoids could not be used for these purposes (data not shown; Gubler and Sherman, 1990).

### DISCUSSION

The present study further elucidates the mechanism of action of liarozole-fumarate by showing its enhancing effect on the antiproliferative activity of naturally occurring ATRA catabolites. These effects could only be properly studied in a steroid- and retinoid-free culture medium supplemented with growth factors. We have previously demonstrated that not only ATRA itself, but also its naturally occurring catabolites, 4-oxo-ATRA and 5,6-epoxy-ATRA, as well as its stereoisomers, 9-*cis*-RA and 13-*cis*-RA, significantly inhibit the proliferation of MCF-7 human breast cancer cells (Van heusden et al, 1998). Liarozole-fumarate, at a concentration of $10^{-6}$ M, enhanced the antiproliferative activity of ATRA and its catabolites, but not that of the stereoisomers of ATRA. As such liarozole-fumarate acts as a versatile inhibitor of the cytochrome P450-dependent ATRA catabolic pathway (Figure 4).

Liarozole-fumarate ($10^{-6}$ M) significantly enhanced the antiproliferative activity of ATRA in MCF-7 cells. This potentiating effect was more than 100-fold at low retinoid concentrations ($10^{-10}$ M to

© Cancer Research Campaign 1998

**Figure 1** Concentration response curves showing the effect of liarozole-fumarate alone (**A**) or in combination with ATRA (**B**), 4-oxo-ATRA (**C**), 5,6-epoxy-ATRA (**D**), 9-*cis*-RA (**E**) and 13-*cis*-RA (**F**) in MCF-7 cells. Cells were cultured for 7 days in the presence of test compounds. Cell proliferation was measured by BrdU incorporation, as described in Materials and methods. Results are presented as means ± s.d. of three (**B, E, F**), four (**A**) or five (**C, D**) experiments. $^{*}P < .01$ and $^{**}P < .001$ vs control cells (Mann–Whitney *U*-test). $^{\#}P < .05$ and $^{\#\#}P < .01$ vs retinoid-treated cells (Mann–Whitney *U*-test). (**A**) ○, Liarozole-fumarate. (**B**) ○, ATRA; ▼, ATRA + 1 μM liarozole-fumarate. (**C**) ○, 4-oxo-ATRA; ▼, 4-oxo-ATRA + 1 μM liarozole-fumarate. (**D**) ○, 5,6-epoxy-ATRA; ▼, 5,6-epoxy-ATRA + 1 μM liarozole-fumarate. (**E**) ○, 9-*cis*-RA; ▼, 9-*cis*-RA + 1 μM liarozole-fumarate. (**F**) ○, 13-*cis*-RA; ▼, 13-*cis*-RA + 1 μM liarozole-fumarate

© Cancer Research Campaign 1998

**Figure 2** Competition between radioactive [$^3$H]ATRA and non-radioactive ATRA ($IC_{50}$ = 129 ± 40 nM) and 5,6-epoxy-ATRA ($IC_{50}$ = 50 ± 28 nM). MCF-7 cells were preincubated for 24 h with $10^{-6}$ M ATRA to induce ATRA catabolism. Concentration response curves for ATRA catabolism were determined using $10^{-7}$ M [$^3$H]ATRA as substrate. Results are presented as means ± s.d. of five experiments. *$P$ < .01 vs control. •$P$ < .05 vs ATRA treatment (Mann–Whitney $U$-test), ○, ATRA; ●, 5,6-epoxy-ATRA

$10^{-9}$ M) in contrast to the tenfold enhancement, as previously demonstrated under growth conditions with culture medium containing untreated FBS (Wouters et al, 1992; Van heusden et al, 1996). The effect of liarozole-fumarate on ATRA metabolism was analysed using HPLC. Only the supernatant was studied. We have previously shown that in the cell extract the same overall metabolite profile can be found (Wouters et al, 1992). Liarozole-fumarate increased the level of RA and concomitantly inhibited the formation of more polar metabolites in a concentration-dependent manner. Together, these findings suggest that liarozole-fumarate enhances the antiproliferative activity of ATRA by inhibiting its catabolism, in agreement with our previous findings (Wouters et al, 1992).

Liarozole-fumarate ($10^{-6}$ M) enhanced the antiproliferative activity of the naturally occurring catabolite 4-oxo-ATRA, confirming that its catabolism is inhibited by liarozole-fumarate. Indeed, Van Wauwe et al (1994) have shown that liarozole-fumarate inhibited the formation of more polar metabolites from 4-oxo-ATRA in hamster liver microsomes. In addition, liarozole-fumarate enhanced the plasma half-life of 4-oxo-ATRA in rats (Van Wauwe et al, 1994). Taken together, these findings suggest that catabolism of 4-oxo-ATRA involves at least one cytochrome P450-dependent enzyme (Roberts et al, 1980; Van Wauwe et al, 1994) that is inhibited by liarozole-fumarate.

The antiproliferative activity of low concentrations of 5,6-epoxy-ATRA was also potentiated by liarozole-fumarate ($10^{-6}$ M). One possible explanation for this synergism is that liarozole-fumarate inhibits the cytochrome P450-dependent catabolism of 5,6-epoxy-ATRA. Although such enzyme(s) are unknown, it is not unlikely that 5,6-epoxy-ATRA is metabolized by the same enzyme(s) as ATRA as 5,6-epoxy-ATRA was a better competitor than ATRA itself in our assay for measuring ATRA catabolism. Although liarozole-fumarate was present at a tenfold higher concentration, i.e. $10^{-6}$ M, the drug was not able to enhance the antiproliferative activity of 5,6-epoxy-ATRA when tested at a concentration of $10^{-7}$ M. These data support the hypothesis that both liarozole-fumarate and 5,6-epoxy-ATRA compete for the same enzyme(s) and that the higher affinity of 5,6-epoxy-ATRA for these enzyme(s) does not allow enhancement.

**Figure 3** Chromatograms illustrating the metabolism of [$^3$H]ATRA in the absence (**A**) or presence of liarozole-fumarate at a concentration of $10^{-6}$ M (**B**) or $10^{-5}$ M (**C**). Cells were incubated for 24 h with $10^{-6}$ M ATRA to induce ATRA catabolism, washed twice and collected. Cells were then incubated for 4 h with $10^{-7}$ M [$^3$H]ATRA and the supernatant was analysed using reverse phase high-performance liquid chromatography (HPLC). I, very polar metabolites; II, intermediate polar metabolites; III, apolar metabolite

**Table 1** Quantitative analysis of [$^3$H]ATRA metabolism in MCF-7 cells

| | Radioactivity (%) | | |
|---|---|---|---|
| **HPLC peaks** | **Control** | **+ $10^{-6}$ M liarozole** | **+ $10^{-5}$ M liarozole** |
| RA (% ATRA, 9-*cis*-RA and 13-*cis*-RA) | 43 (78, 13, 10) | 52 (78, 13, 10) | 73 (82, 10, 9) |
| Metabolites | | | |
| Very polar | 36 | 28 | 10 |
| Intermediately polar | 15 | 12 | 5 |
| Apolar | 6 | 8 | 12 |

**Figure 4** Proposed mechanism of action of liarozole-fumarate. The drug acts as a versatile inhibitor at different steps of the ATRA catabolic pathway. Liarozole-fumarate not only inhibits the catabolism of ATRA, but also of its catabolites 4-oxo-ATRA and 5,6-epoxy-ATRA, thereby enhancing their antiproliferative activity

The ability of ATRA to isomerize in cell culture has been described before (Urbach and Rando, 1994). Liarozole-fumarate ($10^{-6}$ M) had no effect on isomerization of ATRA. The antiproliferative activity of 9-*cis*-RA and 13-*cis*-RA could not be enhanced by liarozole-fumarate ($10^{-6}$ M). In contrast, in vivo liarozole-fumarate has been shown to enhance plasma levels of 9-*cis*-RA (Achkar et al, 1994) and 13-*cis*-RA (Westarp et al, 1994). As RA catabolism may be cell specifically regulated, the most likely explanation for this discrepancy is the absence of cytochrome P450-dependent enzymes that catabolize 9-*cis*-RA and 13-*cis*-RA in MCF-7 cells. In favour of this hypothesis is our finding that 9-*cis*-RA was not converted to polar metabolites in these cultures. Although, we have previously shown that 9-*cis*-RA and 13-*cis*-RA can isomerize to ATRA in MCF-7 cells (Krekels et al, 1997), the fact that their antiproliferative activity was not potentiated by liarozole-fumarate, indicates that their effect on MCF-7 cell proliferation was not due to conversion to ATRA.

We have shown that both 9-*cis*-RA and 13-*cis*-RA compete with ATRA in our assay for measuring ATRA catabolism in MCF-7 cells (Krekels et al, 1997). In this study, we show that liarozole-fumarate was not able to enhance the antiproliferative activity of these stereoisomers. Taken together, these findings suggest that although both stereoisomers are able to bind to ATRA-catabolizing enzyme(s), they are not used as substrates by these enzyme(s) because then an enhancing effect by liarozole-fumarate would be expected. These latter data are in agreement with the observations of Duell et al (1996) in mouse skin.

In conclusion, we can state that liarozole-fumarate is not only able to inhibit the catabolism of ATRA but also that of 4-oxo-ATRA and 5,6-epoxy-ATRA, thereby enhancing their antiproliferative activity. Liarozole-fumarate does not enhance the antiproliferative activity of the stereoisomers 9-*cis*-RA and 13-*cis*-RA in MCF-7 human breast cancer cells, most probably because of the absence of their oxidative catabolism in these cells.

*© Cancer Research Campaign 1998*

Together, these findings extend and confirm the hypothesis that liarozole-fumarate acts as a versatile inhibitor in the ATRA catabolic pathway (Figure 4), and may show potential in circumvention of RA resistance in cancer.

### ACKNOWLEDGEMENTS

We sincerely thank Dr J Van Wauwe for his critical review of the manuscript. We are very grateful to Dr M Klaus (Hoffmann-La-Roche, Basle, Switzerland) for the generous gift of the retinoids. The technical assistance of Hélène Bruwiere and Willy Cools is highly appreciated. The photographic layout by Lambert Leijssen and colleagues is also acknowledged.

### REFERENCES

Acevedo P and Bertram JS (1995) Liarozole potentiates the cancer chemopreventive activity of and the up-regulation of gap junctional communication and connexin43 expression by retinoic acid and β-carotene in 10T1/2 cells. *Carcinogenesis* **16:** 2215–2222

Achkar CC, Bentel JM, Boylan JF, Scher HI, Gudas LJ and Miller WH Jr (1994) Differences in the pharmacokinetic properties of orally administered all-*trans*-retinoic acid and 9-*cis*-retinoic acid in the plasma of nude mice. *Drug Metab Dis* **22:** 451–458

Barua AB, Gunning DB and Olson JA (1991) Metabolism in vivo of all-*trans* [11-$^3$H]retinoic acid after an oral dose in rats. *Biochem J* **277:** 527–531

De Coster R, Wouters W, Van Ginckel R, End D, Krekels M, Coene M-C and Bowden C (1992) Experimental studies with liarozole (R 75251): an antitumoral agent which inhibits retinoic acid breakdown. *J Steroid Biochem Mol Biol* **43:** 197–201

Dijkman GA, Van Moortselaar RJA, Van Ginckel R, Van Stratum P, Wouters L, Debruyne FMJ, Schalken JA and De Coster R (1994) Antitumoral effects of liarozole in androgen-dependent and -independent R3327-Dunning prostate adenocarcinomas. *J Urol* **151:** 217–222

Dolbeare F (1995) Bromodeoxyuridine: a diagnostic tool in biology and medicine, Part I: Historical perspectives, histochemical methods and cell kinetics. *Histochem J* **27:** 339–369

Duell EA, Kang S and Voorhees JJ (1996) Retinoic acid isomers applied to human skin in vivo each induce a 4-hydroxylase that inactivates only *trans* retinoic acid. *J Invest Dermatol* **106:** 316–320

Frolik CA, Roberts AB, Tavela TE, Roller PP, Newton DL and Sporn MB (1979) Isolation and identification of 4-hydroxy- and 4-oxoretinoic acid. In vitro metabolites of all-*trans*-retinoic acid in hamster trachea and liver. *Biochemistry* **18:** 2092–2097

Gubler ML and Sherman MI (1990) Metabolism of retinoic acid and retinol by intact cells and cell extracts. *Methods Enzymol* **189:** 525–530

Hall AK (1996) Liarozole amplifies retinoid-induced apoptosis in human prostate cancer cells. *Anti-Cancer Drugs* **7:** 312–320

Hong WK and Itri LM (1994) Retinoids and human cancer. In *The retinoids: Biology, Chemistry and Medicine*, Sporn MB, Roberts AB and Goodman DS (eds), pp. 597–630. Raven Press: New York

Horwitz KB and McGuire WL (1978) Estrogen control of progesterone receptor in human breast cancer. *J Biol Chem* **253:** 2223–2228

Kizaki M, Ueno H, Yamazoe Y, Shimada M, Takayama N, Muto A, Matsushita H, Nakajima H, Morikawa M, Koeffler HP and Ikeda Y (1996) Mechanisms of retinoid resistance in leukemic cells: possible role of cytochrome P450 and P-glycoprotein. *Blood* **87:** 725–733

Krekels MDWG, Zimmerman J, Janssens B, Van Ginckel R, Cools W, Van Hove C, Coene M-C and Wouters W (1996) Analysis of the oxidative catabolism of retinoic acid in rat Dunning R3327G prostate tumors. *Prostate* **29:** 36–41

Krekels MDWG, Verhoeven A, van Dun J, Cools W, Van Hove C, Dillen L, Coene M-C and Wouters W (1997) Induction of the oxidative catabolism of retinoic acid in MCF-7 cells. *Br J Cancer* **75:** 1098–1104

Lotan R (1980) Effects of vitamin A and its analogs (retinoids) on normal and neoplastic cells. *Biochim Biophys Acta* **605:** 33–91

McCormick AM, Napoli JL, Schnoes HK and DeLuca HF (1978) Isolation and identification of 5,6-epoxy-retinoic acid: A biologically active metabolite of retinoic acid. *Biochemistry* **17:** 4085–4090

Mahler C, Verhelst J and Denis L (1993) Ketoconazole and liarozole in the treatment of advanced prostatic cancer. *Cancer* **71** (suppl.): 1068–1073

Moon RC, Mehta RG and Rao KVN (1994) Retinoids and cancer in experimental animals. In *The Retinoids: Biology, Chemistry and Medicine*, Sporn MB, Roberts AB and Goodman DS (eds), pp. 573–595. Raven Press: New York

Muindi JRF, Frankel SR, Miller WHJr, Jakubowski A, Scheinberg DA, Young CW, Dmitrovsky E and Warrell RPJ (1992) Continuous treatment with all-*trans*-retinoic acid causes a progressive reduction in plasma drug concentrations: implications for relapse and retinoid 'resistance' in patients with acute promyelocytic leukemia. *Blood* **79:** 299–303

Roberts AB, Lamb LC and Sporn MB (1980) Metabolism of all-*trans*-retinoic acid in hamster liver microsomes: oxidation of 4-hydroxy-to 4-keto-retinoic acid. *Arch Biochem Biophys* **199:** 374–383

Smets G, Van Ginckel R, Daneels G, Moeremans M, Van Wauwe J, Coene M-C, Ramaekers FCS, Schalken JA, Borgers M and De Coster R (1995) Liarozole, an antitumor drug, modulates cytokeratin expression in the Dunning AT-6sq prostatic carcinoma through in situ accumulation of all-*trans*-retinoic acid. *Prostate* **27:** 129–140

Smith MA, Adamson PC, Balis FM, Feusner J, Aronson L, Murphy RF, Horowitz ME, Reaman G, Hammond GD, Connaghan GD, Hittelman WN and Poplack DG (1992) Phase I and pharmacokinetic evaluation of all-*trans*-retinoic acid in pediatric patients with cancer. *J Clin Oncol* **10:** 1666–1673

Stearns ME, Wang M and Fudge K (1993) Liarozole and 13-*cis*-retinoic acid antiprostatic tumor activity. *Cancer Res* **53:** 3073–3077

Urbach J and Rando RR (1994) Isomerization of all-*trans*-retinoic acid to 9-*cis*-retinoic acid. *Biochem J* **299:** 459–465

Van Ginckel R, De Coster R, Wouters W, Vanherck W, van der Veer R, Goeminne N, Jagers E, Van Cauteren H, Wouters L, Distelmans W and Janssen PAJ (1990) Antitumoral effects of R 75251 on the growth of transplantable R3327 prostatic adenocarcinoma in rats. *Prostate* **16:** 313–323

Van heusden J, Borgers M, Ramaekers F, Xhonneux B, Wouters W, De Coster R and Smets G (1996) Liarozole potentiates the all-*trans*-retinoic acid-induced structural remodelling in human breast carcinoma MCF-7 cells in vitro. *Eur J Cell Biol* **71:** 89–98

Van heusden J, de Jong P, Ramaekers F, Bruwiere H, Borgers M and Smets G (1997) Fluorescein-labeled tyramide strongly enhances the detection of low bromodeoxyuridine-incorporation levels. *J Histochem Cytochem* **45:** 315–319

Van heusden J, Wouters W, Ramaekers FCS, Krekels MDWG, Dillen L, Borgers M and Smets G (1998) All-*trans*-retinoic acid metabolites significantly inhibit the proliferation of MCF-7 human breast cancer cells in vitro. *Br J Cancer* **77:** 26–32

Van Wauwe JP, Coene M-C, Goosens J, Cools W and Monbaliu J (1990) Effects of cytochrome P-450 inhibitors on the in vivo metabolism of all-*trans*-retinoic acid in rats. *J Pharmacol Exp Ther* **252:** 365–369

Van Wauwe J, Van Nyen G, Coene M-C, Stoppie P, Cools W, Goossens J, Borghgraef P and Janssen P (1991) Liarozole, an inhibitor of retinoic acid metabolism, exerts retinoid-mimetic effects in vivo. *J Pharmacol Exp Ther* **261:** 773–779

Van Wauwe J, Coene M-C, Cools W, Goosens J, Lauwers W, Le Jeune L, Van Hove C and Van Nyen G (1994) Liarozole-fumarate inhibits the metabolism of 4-keto-all-*trans*-retinoic acid. *Biochem Pharmacol* **47:** 737–741

Warrell RPJr (1993) Retinoid resistance in acute promyelocytic leukemia: new mechanisms, strategies, and implications. *Blood* **82:** 1949–1953

Warrell RPJr, de The H, Wang ZY and Degos L (1993) Acute promyelocytic leukemia. *N Engl J Med* **329:** 177–189

Westarp ME, Westarp MP, Bollag W, Bruynseels J, Biesalski H, Grossmann N and Kornhuber H-H (1994) Effect of six retinoids and retinoic acid catabolic inhibitor liarozole on two glioblastoma cell lines, and in vivo experience in malignant brain tumor patients. In *Cancer Treatment – an Update*, Banzet P, Holland JF, Khayat D and Weil M (eds), pp.590–598. Springer: Paris

White JA, Guo Y-D, Baetz K, Beckett-Jones B, Bonasoro J, Hsu KE, Dilworth FJ, Jones G and Petkovich M (1996) Identification of the retinoic acid-inducible all-*trans*-retinoic acid 4-hydroxylase. *J Biol Chem* **271:** 29922–29927

Wouters W (1994) Retinoid metabolism and its inhibition by liarozole-fumarate. *Ann Oncology* **5:** S45–S47

Wouters W, van Dun J, Dillen A, Coene M-C, Cools W and De Coster R (1992) Effects of liarozole, a new antitumor compound, on retinoic acid-induced inhibition of cell growth and on retinoic acid metabolism in MCF-7 human breast cancer cells. *Cancer Res* **52:** 2841–2846

© Cancer Research Campaign 1998